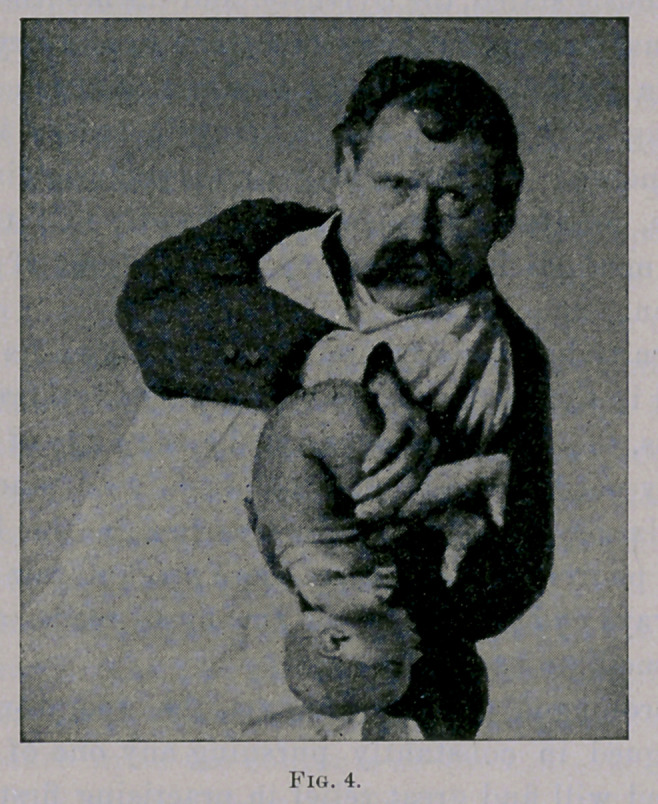# Establishing a New Method of Artificial Respiration in Asphyxia Neonatorum1Read before the New York Academy of Medicine, February 2, 1893, and published in the *Medical Record*, March 11, 1893. Cuts kindly loaned by the *Medical Record*.

**Published:** 1894-06

**Authors:** J. Harvie Dew

**Affiliations:** 252 West Fifty-fourth Street; New York


### ESTABLISHING A NEW METHOD OF ARTIFICIAL RESPIRATION IN ASPHYXIA NEONATORUM.[1]

By J. HARVIE DEW, M. D., New York.

(*With Four Illustrations.*)

### DESCRIPTION OF METHOD.

My directions for its practice are: To grasp the infant with the left hand, allowing the neck to rest between the thumb and forefinger, the head falling far over backward, straightening the mouth with the larynx and trachea, thereby serving to raise and hold open the epiglottis (as demonstrated by Benjamin Howard

1. Read before the New York Academy of Medicine, February 2, 1893, and published in the *Medical Record*, March 11, 1893. Cuts kindly loaned by the *Medical Record*.

in his excellent article, A New and Only Way of Raising the Epiglottis, *British Medical Journal*, November, 1888). The upper portion of the back and scapulæ resting in the palm of the hand, the other three fingers to be inserted in the axilla of the baby's left arm, raising it upward and outward. (See Fig. 1.)

Then, with the right hand, if the baby is large and heavy, grasp the knees in such a way as to hold them with the right knee resting between the thumb and forefinger, the left between the fore and middle fingers. This position will allow the back of the thighs to rest in the palm of the operator's hand. If the infant is small and light, it will be found more convenient and easier to

FIG. 1.

hold it in the same way by the ankles instead of the knees, allowing the calves instead of the thighs to rest in the palm of the hand.

The next step is to depress the pelvis and lower extremities, so as to allow the abdominal organs to drag the diaphragm downward, and with the left hand to gently bend the dorsal region of the spine backward. This enlarges the thoracic cavity and produces inspiration. (See Fig. 2.)

Then, to excite expiration, reverse the movement, bringing the head, shoulders and chest forward, closing the ribs upon each other, and at the same moment bring forward the thighs, resting them upon the abdomen. This movement arches the lumbar region backward, and so bends the child upon itself as to crowd together the contents of the thoracic and abdominal cavities, resulting in a most complete and forcible expiration. (See Fig. 3.)

While this movement is a powerful one, the operator can, by his manipulations, accomplish it without shock and render it as gentle as he pleases. . . . . . . . . . . .

### EXPLANATION OF METHOD.

At birth an asphyxiated infant is perfectly limp and flexible. Its muscles are like so many wet rags, and offer no resistance till stretched out to near or about the limit of their elasticity. In

FIG. 2.

the Sylvester method the ribs are not lifted till the pectoral muscles have been put well upon the stretch, for the accomplishment of which the arms must be forcibly pulled upward.

When this is done, the chest cavity is increased laterally, and the diaphragm is flattened out, pressing the abdominal organs to some extent downward, thus serving, in a measure, to increase the cavity vertically. This produces the suction which every one recognizes, and which has made this method, up to date, the most universally known and adopted.

My method accomplishes exactly the same results in a different way. To understand how it is done, let us consider for a moment the anatomical structure of the chest walls. These walls are supported by, and have their fixed point in the attachment of the ribs to the dorsal vertebræ. They are composed mainly of the ribs, their cartilages, the sternum and the intercostal and pectoral muscles.

The muscles, as stated, offer no resistance and no assistance, except for traction.

The ribs constitute not only the most prominent structure in the formation of the chest walls, but their movements are essentially important in any effort artificially or naturally to draw air

Fig. 3.

into the lungs. It is upon their peculiar arrangement, formation and attachments that the active inspiratory movement of my method depends.

They are twelve in number on each side, and are separated from each other at well-defined distances. They vary in both length and shape from the first to the twelfth. They can be made to very closely approximate, if not to overlap each other, and are capable of as wide a separation as the elasticity of the intercostal muscles will permit. They terminate at the sternum in flexible cartilages, which vary in length and render them very movable.

Posteriorly they have almost a fixed attachment. Their heads are closely bound by a strong ligamentous union to the bodies of the dorsal vertebræ, while their tubercles, located nearly an inch from their heads, are bound with equal firmness to the lateral processes of the same vertebræ. Only a slight rotatory motion exists at this articulation, which, together with the peculiar shape of the ribs and the flexibility of their anterior attachments, enables the normal inspiratory act to be performed, the ribs at each effort being drawn upward and outward.

Now comes the important fact I wish to impress. It is, that in my method of artificial respiration, owing to the firm attachment of the ribs to the bodies and processes of the vertebræ, as soon as the dorsal region is curved backward and the relative position of the bodies and transverse processes is changed, the ribs and their intercostal muscles open out like the segments of a fan, and, at the same time, owing to their peculiar shape, all of the bodies of the longer ribs are forced outward and the diaphragm is flattened. Thus, both the lateral and vertical diameters of the thoracic cavity are increased.

How much air is actually drawn in and how much reflex action is excited by the inspiratory effort of this or any other method in the first few movements it is difficult, from a clinical standpoint, to determine, but after keeping up the operation for a few moments, in any favorable case, it will be easy to recognize unquestioned evidences of suction.

The infant whose photograph I herewith exhibit was born a little before, and died a short while after, 7 P. M. I was not present at its birth, but reached the bedside a few minutes after death. The photographs were taken at 11 A. M., sixteen hours after death. I then performed my method of artificial respiration, and was able to force air in and out of the lungs with each movement. The evidence was made positive by a very audible sound, excited by the escape of air at each expiratory effort.

It is very frequently, if not usually the case, when resuscitating an infant, that a decided grunt is heard with the expiratory movement after once the introduction of air has been established.

The expiratory movement in this method is one of its most perfect and advantageous features. Indeed, I believe that a complete expiration is, for at least two reasons, of equal, if not greater importance than that of inspiration: 1st, because if one cubic inch, or any given amount of air is drawn in, it is most desirable that all of it shall be forced out in the movement that follows; 2d, because the expiratory effort in artificial respiration should not serve only for the expulsion of air, but should at the same time be a means of improving and hastening the general circulation.

If the thoracic cavity is thoroughly but gently compressed, the heart and large blood-vessels will be unloaded in the direction of least resistance. This, of course, must be forward and in the right direction, as the cardiac aortic and pulmonary valves will open for its forward and close upon its backward flow. The accomplishment of this result with each expiration cannot be otherwise than most beneficial to the sluggish circulation of an asphyxiated infant. The Schultze method fills this requisite, as pointed out by Dr. Lusk in his article upon this subject; but the action is too violent and cannot be regulated with gentleness. The Sylvester method and its modifications, the mouth-to-mouth insufflation, and inflation by catheterization, are all deficient in this particular: they, each of them, depend for their expiratory movement on lateral pressure over the lower ribs, upon the epigastrium, or both together. This plan of expiration is objectionable because: 1. It does not expel all of the air from the lungs, if any has been drawn in. 2. It causes the center and posterior portion of the flabby diaphragm to descend, thereby increasing the vertical diameter of the chest cavity. 3. It produces but slight, if any, pressure upon the heart and large blood-vessels which occupy the mediastinum; certainly not sufficient pressure to be of any material benefit to the circulation.

In the expiratory movement of my method, when the shoulders and chest are brought forward, and at the same moment the thighs are made to rest upon the abdomen, including the epigastric region, the pressure upon the contents of the thoracic cavity can be made as forcible as the operator thinks best. The ribs are crowded upon each other, closing up the intercostal spaces, and the organs of the abdomen are pushed upward upon the diaphragm so as to diminish the vertical diameter as much as it is possible to do. By these combined forces the expulsion of air is complete, and the desired effect upon the heart and large blood-vessels is most favorably secured.

### A METHOD MUST BE SELECTED.

Every obstetrician who finds that he has delivered an asphyxiated infant proceeds at once to excite the respiratory act by reflex stimulus. To do this he moves the infant from side to side, spanks it, sprinkles water upon it, and possibly dips it alternately into hot and cold water ; but when the asphyxia is too profound to be thus relieved, he is forced to resort to some one of the many methods of artificial respiration.

Of the established methods Sylvester's and the plan of mouth-to-mouth inflation are probably by far the most universally adopted, next that of Schultze, then catheterization and insufflation, and finally the individual plans not commonly known. One or more of these methods must be resorted to by every practitioner. Hence, it is a matter of unquestionable importance to be able to select the best among them, not only for individual use but for instruction in our schools of medicine.

As previously stated, the Sylvester and the mouth-to-mouth plan offer good inspiratory but very imperfect expiratory movements. The Schultze method, though very efficient, is often inconvenient, is too chilling to the infant, and in many instances is too violent in its movements. Catheterization and insufflation is not easy, and is, as a rule, unsafe in inexperienced hands. Of the individual and private methods I have nothing to say except of the one under consideration.

In maternity hospitals where the obstetrician is offered every facility, and in the homes of the wealthy where there are so many conveniences, the difference between the methods of artificial respiration may not be a question of so much importance; but in that very much larger class of cases occurring in the homes of the middle and poorer people, where there are but few conveniences, he must always endeavor to select the most ready and favorable plan for immediate use.

In any prolonged case of asphyxia, the operator will become greatly fatigued in constantly pursuing any one of the methods proposed, and will find great relief in practising first one plan and then another.

### ADVANTAGES OF THIS METHOD.

I claim for my method the following facts and advantages:

1. That it is most efficient in all cases where artificial respiration, in asphyxia neonatorum, is indicated.

2. That years of experience have served to prove to others, as well as myself, its unquestioned value.

3. That it can be practised with ease and readiness to the operator.

4. That its movements are easy and can be quickly resorted to at any moment and anywhere.

5. That while its inspiratory movement will be found, by experience at the bedside, to be as efficient as that of other methods, the expiratory movement is far more complete and satisfactory than in any of them.

6. That nearly, or about all, of the air drawn in can be expelled.

7. That, owing to the force and at the same time to the absolute control which the operator has over the expiratory movement, he is able to compress the contents of the thoracic cavity to just exactly that degree deemed by him wisest and best, thereby favoring and hastening the general circulation.

Fig. 4.

8. That this method can be employed before the cord is cut, when it seems important to save as much blood as possible to the infant.

9. That the operator can sit or move from place to place about the room, greatly to his relief from fatigue, still continuing the respiratory movements.

10. That, if thought best, the movements can be kept up while the infant is immersed up to its chin in hot water.

11. That by elevating the buttocks and depressing the head and shoulders, the expulsion of mucus can be effected, as in the Schultze method. (See Fig. 4.)

12. That for alternating with Sylvester's and other methods it possesses peculiar advantages, affording great relief to tiresome positions in protracted cases.

13. That it possesses all of the advantages of the Schultze method and none of its disadvantages.

14. That the method is prompt, reliable, easy to perform and perfectly safe.

252 West Fifty-fourth Street.

## Figures and Tables

**Fig. 1. f1:**
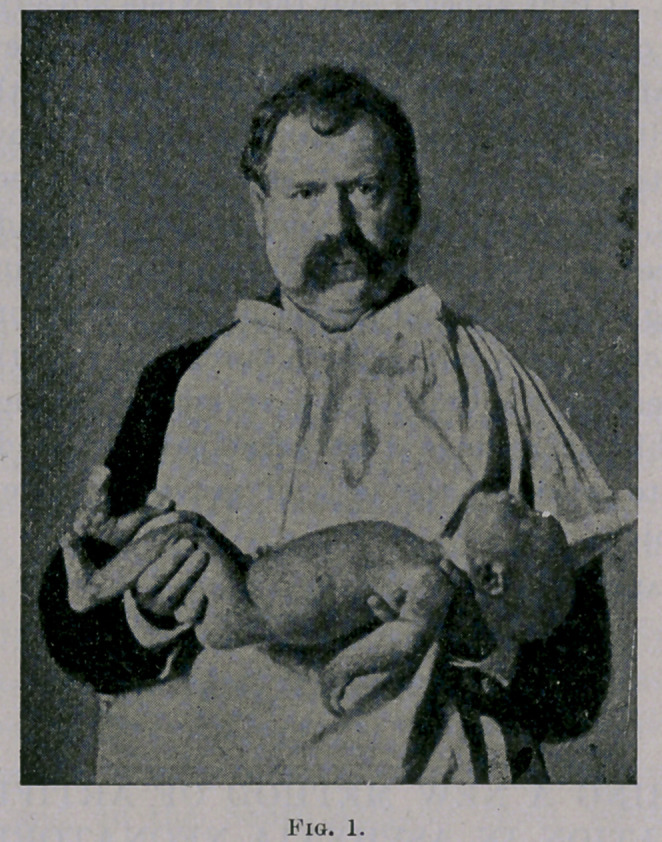


**Fig. 2. f2:**
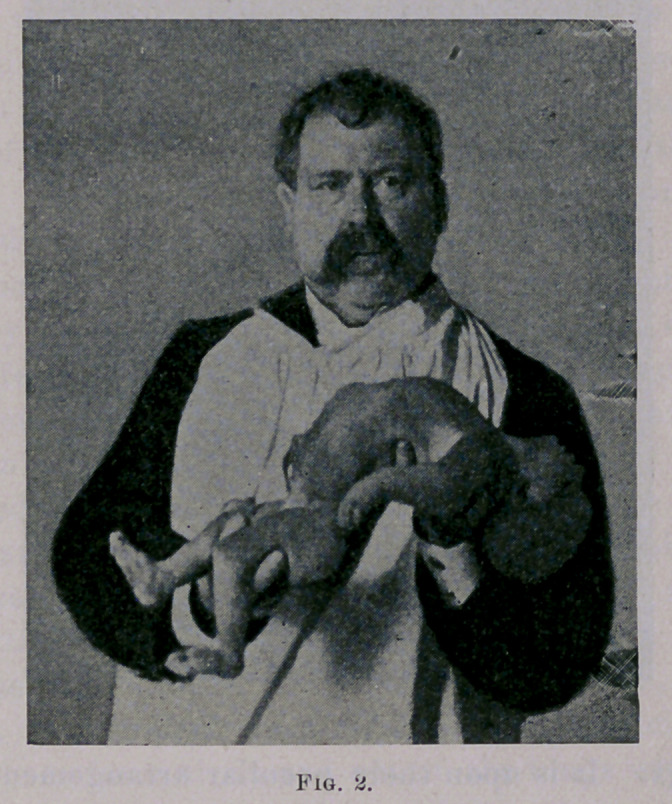


**Fig. 3. f3:**
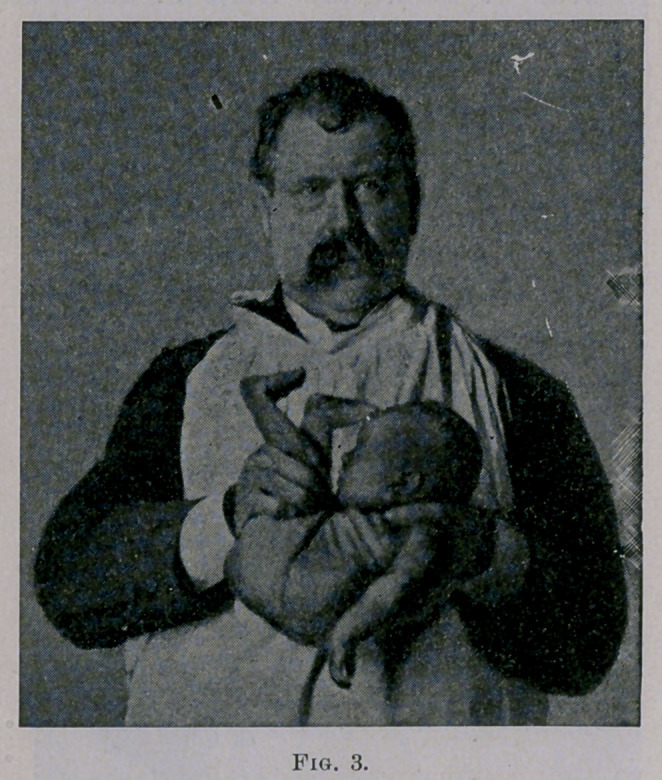


**Fig. 4. f4:**